# The Role of Postoperative Radiotherapy in the Management of Dermatofibrosarcoma Protuberans: A Multidisciplinary Systematic Review

**DOI:** 10.3390/jcm13061798

**Published:** 2024-03-21

**Authors:** Bruno Fionda, Antonella Loperfido, Alessandro Di Stefani, Valentina Lancellotta, Andrea Paradisi, Martina De Angeli, Simone Cappilli, Ernesto Rossi, Anna Amelia Caretto, Tiziano Zinicola, Giovanni Schinzari, Stefano Gentileschi, Alessio Giuseppe Morganti, Agata Rembielak, Ketty Peris, Luca Tagliaferri

**Affiliations:** 1 U.O.C. Radioterapia Oncologica, Dipartimento di Diagnostica per Immagini, Radioterapia Oncologica Ed Ematologia, Fondazione Policlinico Universitario Agostino Gemelli IRCCS, 00168 Rome, Italy; bruno.fionda@policlinicogemelli.it (B.F.);; 2 Otolaryngology Unit, San Camillo Forlanini Hospital, 00152 Rome, Italy; 3 U.O.C. di Dermatologia, Dipartimento di Scienze Mediche e Chirurgiche, Fondazione Policlinico Universitario Agostino Gemelli IRCCS, 00168 Rome, Italy; 4 Dermatologia, Dipartimento di Medicina e Chirurgia Traslazionale, Università Cattolica del Sacro Cuore, 00168 Rome, Italy; 5 Medical Oncology Unit, Fondazione Policlinico Universitario Agostino Gemelli IRCCS, 00168 Rome, Italy; 6 Dipartimento Universitario di Medicina e Chirurgia Traslazionale, Università Cattolica del Sacro Cuore, 00168 Rome, Italy; 7 Unità di Chirurgia Plastica, Dipartimento Scienze della Salute della Donna e del Bambino e di Sanità Pubblica, Fondazione Policlinico Universitario Agostino Gemelli IRCCS, 00168 Rome, Italy; 8 Division of Radiation Oncology, IRCCS Azienda Ospedaliero-Universitaria di Bologna, 40138 Bologna, Italy; 9 Department of Radiation Oncology, The Christie NHS Foundation Trust, Manchester M20 4BX, UK; 10 Division of Cancer Sciences, Faculty of Biomedicine and Health, School of Medical Sciences, The University of Manchester, Manchester M13 9PL, UK; 11 Istituto di Radiologia, Università Cattolica del Sacro Cuore, 00168 Rome, Italy

**Keywords:** dermatofibrosarcoma protuberans, DFSP, radiotherapy, radiation therapy

## Abstract

**Background**: Dermatofibrosarcoma protuberans (DFSP) is a superficial soft tissue sarcoma, and surgical excision is the first-line treatment. The aim of this systematic review is to provide an update about the current indications and clinical results regarding the use of postoperative radiotherapy in DSFP, considering both adjuvant and salvage setting. **Methods**: We conducted a systematic literature review using the main scientific database, including Cochrane library, Scopus, and PubMed, for any relevant article about the topic, and we considered all available papers without any time restriction. **Results**: Twenty-two papers, published between 1989 and 2023, were retrieved and considered eligible for inclusion in this review. Regarding the fractionation schedules, most authors reported using standard fractionation (2 Gy/die) with a wide total dose ranging from 50 to 70 Gy. The local control after postoperative radiotherapy was excellent (75–100%), with a median follow-up time of 69 months. **Conclusions**: After the primary surgical management of DFSP, postoperative radiotherapy may either be considered as adjuvant treatment (presence of risk factors, i.e., close margins, recurrent tumours, aggressive histological subtypes) or as salvage treatment (positive margins) and should be assessed within the frame of multidisciplinary evaluation.

## 1. Introduction

Dermatofibrosarcoma protuberans (DFSP) represents a superficial soft tissue sarcoma that involves the dermis, subcutaneous fat and, in rare cases, muscle and fascia [1].

It was first described in 1924 by Darier and Ferrand [2] as progressive and recurrent dermatofibroma, and was subsequently named DFSP by Hoffmann [3].

The incidence is 0.8–4.5 cases per one million persons and, despite being the most common cutaneous sarcoma, it constitutes less than 0.1% of all malignancies, and about 1.0% of all soft tissue sarcomas worldwide [4]. It mainly affects young and middle-aged adults, with a peak of incidence between the second and fifth decade of age [5].

Clinically, DFSP presents as a slow-growing, firm, multilobular nodule or plaque that ranges in colour from flesh-coloured to red and has irregular margins on the trunk (50%), preferentially on the shoulder girdle, upper and lower limbs (30–40%), and head-neck area (10–15%) [6,7,8]; rarely, DFSP has been reported to occur on toes [9,10,11,12,13], the scalp [14], breasts [15,16,17], and vulva [18].

In most patients, the age at diagnosis is between 20 to 59 years; however, 10–15% of lesions develop in children and adolescents. A definitive diagnosis requires a histological examination of a skin biopsy showing diffuse infiltration of the dermis and the subcutaneous fat by densely packed, spindle-shaped, CD34-positive tumour cells, arranged in a predominantly storiform pattern [19,20,21]. Less common histologic subtypes of DFSP are comprised of mixoid, pigmented (Bednar tumour), giant cell fibroblastoma, granular cell, sclerosing, atrophic, and fibrosarcomatous variants [22].

Dermoscopic features of DFSP have been reported so far in both case series and single case reports. The findings associated with this sarcoma included a pink-colored background, and structureless depigmented areas, and vessels (linear and arborizing); in addition, shiny white streaks and a fine pigment network were observed, although less specific for this condition [23]. In a large series, the median number of dermoscopic features was four per lesion, and the most common were a delicate pigmented network (87%), vessels (80%), structureless light brown areas (73%), shiny white streaks (67%), pink background coloration (67%), and structureless hypo- or depigmented areas (60%) [24].

DFSP is a locally aggressive tumour with a significant rate of local recurrence depending on treatment modalities (0–40%). It has a low metastastic potential; the 10-year disease-specific survival (DSS) is approximately 99% [25]. Fibrosarcomatous transformation within DFSP represents a rare event, which is characterized by exhibiting more aggressive behaviour, a higher rate of local recurrence, and distant metastases after surgery, as compared to the other histological subtypes [26]. Rare cases with lung, bone, or locoregional lymph nodes metastases have been described [27].

The first-line treatment of DFSP is surgical excision [28]. Mohs’ micrographic surgery (MMS), when available, might be preferrable, while a lateral safety margin of 3 cm is recommended using standard 2D surgical excision [29].

The role of integrated approaches, such as radiotherapy (RT) and/or systemic treatment (i.e., imatinib mesylate), has been reported, both in the neoadjuvant and adjuvant setting, as well as for the definitive treatment in patients not eligible for surgery [30,31,32].

The aim of this systematic review is to provide an update about current indications and clinical results regarding the use of postoperative RT in DSFP considering both adjuvant and salvage setting.

## 2. Materials and Methods

**Figure 1:**
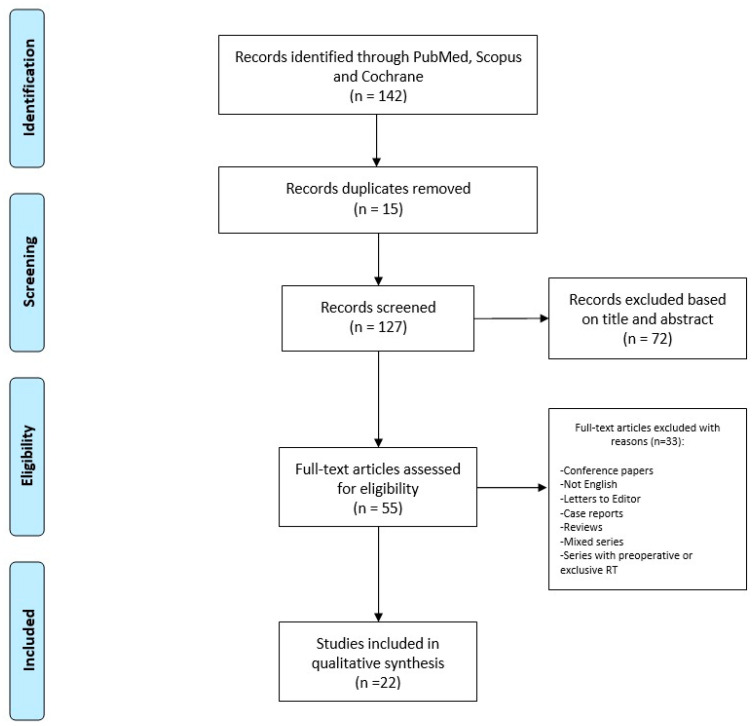
Search strategy.

We conducted the search strategy in accordance with the Preferred Reporting Items for Systematic Reviews and Metanalysis (PRISMA) guidelines for systematic reviews, as reported in Figure 1.

Research was conducted in the main scientific database, including Cochrane library, Scopus, and PubMed, for any relevant article about the topic, and we considered all available papers starting from the inception of databases to June 2023. Several combinations of key terms were used to perform the search, including “Dermatofibrosarcoma” or “Dermatofibrosarcoma Protuberans”, and “Radiotherapy” or “Radiation Therapy”.

The inclusion criteria were original series focusing on DFSP and post-operative RT, with a retrospective and prospective setting. Conference papers, articles not in the English language, letters to the Editor, single case reports, reviews, articles including mixed series with other histologies, and series where RT was performed pre-operatively or as the only therapy were not included.

Two independent authors, a radiation oncologist (BF), and a surgeon (AL) made the study selection through screening titles and full abstracts retrieved from the searches, with the aim of identifying articles that met the inclusion criteria. Subsequently, all the articles were retrieved for full-text analysis to assess eligibility (SC, MD, TZ, AAC, and ER). In cases of uncertainties about their inclusion in the present review, articles were additionally examined by another team composed of expert specialists (ADS, AP, VL, and GS) who performed an independent check. Finally, a multidisciplinary Master committee, composed by senior experts in external beam RT and interventional RT, senior dermatologists, a senior surgeon, and a senior medical oncologist (SG, AGM, KP, and LT), conducted an additional review before the final approval of the definitive version.

## 3. Results

Twenty-two papers, published between 1989 and 2023, were retrieved and considered eligible for inclusion in this review. All investigated studies had a retrospective design and were published by North American, European, or Asian authors. Overall, 994 patients were included and reported upon in the present systematic review. A full and comprehensive list of all the collected variables is provided in Table 1.

The mean age of the patients who were referred for postoperative RT after the surgical removal of DFSP was 40 years (range 11–48) with a slight male prevalence (59%).

The most common site invovled was the trunk, followed by the extremities and the head-neck area.

The average size of the larger diameter of DFSP was 14 cm (with lesions up to 25 cm).

The surgical procedure performed in almost all of the patients before postoperative RT was WLE and, in particular, most series included patients treated both in the adjuvant (R0) and in the salvage (R1) setting; the most commonly reported reasons for adjuvant treatment included inadequate margins, multiple recurrences, or fibrosarcomatous differentiation.

There was a rather large amount of radiotherapy techniques used during the time period of the included articles’ publishing dates. Some authors reported using an electron beam or 2D techniques, whereas more recent papers used 3D and volumetric techniques; in a few cases they also utilized interventional radiotherapy (brachytherapy).

Regarding the fractionation schedules, most authors reported using standard fractionation (2 Gy/die) with a wide total dose ranging from 50 to 70 Gy.

The local control after RT was excellent (75–100%), with a median follow-up time ranging from 1 year to approximately 11 years (median: 69 months).

Only a few authors reported the side effects of their RT treatment; for example, Castle et al. reported breast implant asymmetry, oedema, xerostomia, osteoradionecrosis, and soft-tissue necrosis [37]; Sun et al. found only fibrosis or telangiectasia [43]; Suit et al. encountered atrophy and telangiectasia [47]; and Marks et al. encountered transient skin reaction, wound breakdown, and graft failure [48]. In the following paragraph, we describe, in detail, the result of the single studies described in Table 1.

### Single Studies Results

Dai et al. [1] conducted a retrospective study of forty-nine patients with head and neck dermatofibrosarcoma protuberans. They collected patients affected by HNDFSP who had received surgical treatment. Eight patients (16.3% of all cases) were treated by postoperative radiotherapy (60 Gy), resulting in a remarkable effect on local disease control. Tumour size, patients’ age, and negative margins with enough safety width constituted the main independent factors affecting disease-free survival. The authors concluded that, despite HNDFSP being a rare disease, RT could improve the prognosis of those patients who were experiences significant challenges, as well as a worse prognosis, in using the current treatment strategies.

Mareş et al. [27] retrospectively reported about seven patients who were affected by DFSP, of whom four were males and three were females. They had a mean age of 38.2 years. All patients underwent surgical treatment with wide local excision, with a margin of 3 cm in five patients and 2 cm in the other two. Adjuvant radiotherapy (50–60 Gy doses) was performed in three patients (42.9% of all cases) who presented with deep fascial invasion after deep margin assessment. The authors concluded by emphasizing the importance of the multidisciplinary approach to good treatment, due to the high rate of DFSP recurrence.

Wang et al. [17] performed a retrospective analysis that included six breast DFSP patients (five female and one male with a mean age of 29.7 years). All patients underwent surgical treatment; specifically, five patients underwent a preoperative excisional biopsy folled by a wide local excision, while one patient underwent a wide local excision without a preoperative biopsy because of clinical suspicion of disease recurrence. Two patients were treated by RT. With a median follow-up of 36 months, all six patients survived without metastasis or recurrence. The authors concluded that DFSP of the breast is characterized by similar clinical features to DFSP at other sites, and the risk of recurrence can be reduced through surgical excision with margins of at least 2 cm.

Du et al. [33] assessed the role of postoperative radiotherapy in DFSP management through analyzing a total of 184 patients (140 male and 44 female) with a median age of 41. The most common site involved was the trunk (71.7%), followed by the head and neck (17.4%), and the extremities (10.9%). All patients underwent surgical resection, and 44 of them (23.9%) were treated with postoperative radiotherapy (50–66 Gy doses). The median follow-up time was 58 months, and the 3-year disease-free survival (DFS) was 94.6%. The authors concluded that postoperative RT can potentially improve DFS for high-risk DFSP. Moreover, the authors affirmed that Ki-67 might become a prognostic molecular marker in those patients.

Tsai et al. [22] described thirteen patients with DFSP (six male and seven female patients, median age of 11 years). In seven patients the lesion involved the trunk, in three cases the extremities were affected, in two patients the back was affected, and finally, in one case, the involved site was the head. All patients underwent a surgical wide excision, and three patients underwent adjuvant RT. The mean follow-up period was 129 months and there was no tumor recurrence reported.

Williams et al., 2014 [34] included fourteen patients with DFSP who were treated with radiotherapy (seven male and seven female, median age: 42 years). Regarding tumor location, in eight cases the site involved was the head and neck, in five patients the lesion affected the extremities and, finally, one patient had the lesion on the trunk. Thirteen out of fourteen patients underwent surgery before RT. The median follow-up was 126 months, and local control was described in 85.7% of cases.

Hamid et al., 2013 [35] described thirty-six patients diagnosed with DFSP (twenty-six male patients and ten female patients with a mean age of 38 years), of whom thirty patients underwent surgical treatment and postoperative adjuvant radiotherapy, and six patients were treated with radiotherapy alone. The head and neck were involved in three cases, the trunk was affected in twenty cases, twelve patients had the lesion in the extremities and, in one case, the external genitalia were involved. The median follow-up duration was 68 months and local control was achieved in 80% of patients.

Uysal et al., 2013 [36] retrospectively evaluated twenty-eight patients treated with radiotherapy for DFSP, of whom twenty-five subjects underwent postoperative adjuvant radiotherapy while three patients were treated with only radiotherapy. Eighteen patients were male and ten were female, with a median age of 26 years. Tumor location was 64% to the extremities, 22% to the trunk, and 14% to the head and neck district. The dose of RT delivered was between 50 and 70 Gy, and patients were followed for an average of 81 months. Regarding patients undergoing adjuvant surgery and radiotherapy, the five-year relapse-free survival (RFS) of the twenty patients treating with RT after wide excision was 89.6%, and the 5-year RFS of the five subjects undergoing adjuvant RT after limited excision was 74%, demonstrating a statistically significant difference between limited excision + RT and wide excision + RT groups.

Castle et al., 2013 [37] retrospectively collected fifty-three DFSP patients who were treated with surgery and preoperative or postoperative RT. Specifically, forty-six (87%) of the fifty-three patients were treated with postoperative RT (60–66 Gy). 57% of all cases were male and 43% were female, with a mean age of 41 years. In 36% of cases the lesion was on the trunk, in 21% of patients the tumor occurred on the scalp, in 15% of cases the lesion involved the head-neck district and, finally, in 28% the lesion affected the extremities. The median follow-up duration was 78 months and local control was achieved in 90% of patients.

Palmerini et al., 2012 [38] described a total of forty patients, 55% male and 45% female with a mean age of 43 years. The tumor involved the limbs in 60% and the trunk in 40%. Ninety percent of all patients received previous surgical treatment, and 27% underwent adjuvant RT. With a median follow-up of 49 months, the local control was achieved in 77% of patients.

Fields et al., 2011 [31] identified 244 patients treated for DFSP (50% male, 50% female, mean age 42 years). Extremities were the most commonly involved site (72%), followed by the trunk (31%), the head and neck district (14%), and other unspecified sites (2%). All patients underwent the wide surgical excision and 14 patients (5.7%) received posoperative RT. The median duration of follow-up was 50 months and, in all these cases, local control was achieved.

Archontaki et al., 2010 [39] reported the results of the treatment of sixteen patients affected by DFSP (nine females and seven males, with a mean age of 41 years). Tumour localization was to the trunk and proximal extremities in nine patients, to the lower extremities in two cases, and to the head-neck district in five patients. Primary treatment consisted of a surgical approach, and adjuvant RT was provided in two patients with local recurrence and one patient with massively extensive disease (18.75% of all cases). The median follow-up was about 44 months and all patients remained free of disease recurrence.

Heuvel et al., 2010 [40] described thirty-eight patients with DFSP (25 males and 13 females, with a median age of 38 years) in whom treatment consisted of surgery and, in cases of marginal or positive resection margins, adjuvant RT. The tumour involved the head and neck district in 16.7% of cases, the trunk in 66.6% of cases, and the extremities in 16.7% of patients. Adjuvant RT (50–70 Gy) was applied for eight patients (21%). The median follow-up was 89 months and the local control was achieved in 87.5% of patients.

Dagan et al., 2005 [41] reported a study of ten patients with DFSP that were treated with surgery and postoperative RT. There was an equal distribution between men and women and the mean age was 39 years. In six patients the lesion affected the head-neck district, in three patients the extremities and, finally, one patient had the tumor on the trunk. Postoperative RT was administered in doses ranging from 59.4 to 65 Gy, the local control was achieved in 90% of patients, and median duration of follow-up was 95 months.

DuBay et al., 2004 [42] described 62 patients with DFSP who underwent surgery. Thirty-nine subjects (63%) were female, twenty-three cases (37%) were male, and the mean age was 42 years. In forty-eight cases (76%) the lesion was located on the trunk or extremities, while in 15 cases (24%) the tumour was located on the head and neck region. Postoperative RT was administered in 5% of cases, with a 100% of local control, and median duration of follow-up was 53 months.

Sun et al., 2000 [43] collected 35 patients with DFSP that were treated with surgery with or without RT. The patients were 24 males and 11 females, with a median age of 37 years. The tumour locations were found on the trunk in 21 cases, the extremities in eight cases, and the head and neck district in six cases. Ten patients (28.5%) were treated with postoperative RT with a dose ranging from 46 to 68 Gy. With a median follow-up of 50 months, 81.8% of patients achieved local control.

Stojadinovic et al., 2000 [44] described 33 patients affected by head and neck DFSP, including 17 women and 16 men, with a mean age of 39 years. The anatomical distribution of tumours included 14 cases of scalp involvement, 6 neck injuries, and 13 face lesions. All patients underwent surgical treatment, and four patients (12.1% of cases) received adjuvant RT in doses ranging from 60 to 66 Gy. At median follow-up of 82 months, 75% of patients achieved local control.

Mentzel et al., 1998 [26] analysed 41 patients affected by the fibrosarcomatous variant of dermatofibrosarcoma protuberans (FS-DFSP), including 19 women and 22 men, with a mean age of 48 years. In twenty-five cases the lesion affected the trunk, in ten cases the tumour involved the extremities, and in five patients the lesion affected the head and neck district. Finally, there was one case in which the anatomical site of the tumour was unknown. In forty patients the tumour was surgically removed, while in one case the exact treatment was unknown. In addition, adjuvant RT was administered in three cases (7.4% of cases). The median follow-up was 90 months.

Ballo et al., 1998 [45] described 19 patients with DFSP who were treated with surgery and adjuvant RT to doses of 50–60 Gy. The patients included 12 males and 7 females with a median age of 40 years. Regarding anatomical distribution, the tumour affected the trunk in eight cases, the head and neck region in seven cases, and the extremities in four cases. At median follow-up of 72 months, 94.7% of patients achieved local control.

Haas et al., 1997 [46] performed a retrospective analysis on 38 patients affected by DFSP and who were surgically managed. A total of 22 females and 16 males with a mean age of 39 years were included. In 13 patients the tumour affected the extremities, in 11 patients the abdomen, in 6 patients the back, and in 5 patients the head and neck. RT was performed in 44.7% of patients in doses of 50–66 Gy, the median follow-up was 68 months, 82.4% of patients achieved local control.

Suit et al., 1996 [47] reported outcomes of eighteen patients affected by DFSP and who were treated with RT to doses of 50–67 Gy. In 15 patients, treatment included the combination of radiotherapy and surgery, while 3 patients received RT alone. Eleven patients were males and seven patients were females, with a mean age of 46 years. Regarding the anatomical distribution, in ten cases the lesion affected the head and neck, in five cases the trunk was affected, and in three patients it was the extremities. The median follow-up was 86 months, 83.3% of patients achieved local control.

Marks et al., 1989 [48] described ten patients with DFSP, including four women and six men, with a mean age of 44.4 years. The lesion involved the head and neck in five cases, in four cases the lesion affected the trunk, and in one case the tumour occurred to the extremities. RT was administered in 70% of patients, and the median follow up was 12 months, 90% of patients achieved local control.

## 4. Discussion

DFSP accounts for around 1–5% of all soft tissue sarcomas that affect adults, and account for 18% (the most common) of all cutaneous soft tissue sarcomas [49]. The most frequent localizations are on the trunk and extremities, while the head-neck region is less frequently involved [42].

Molecular studies have improved our knowledge on DFSP pathophysiology. Translocation involving chromosomes 17q22 and 22q13 leads to a fusion protein that promotes the continuous activation of PDGF receptor beta (PDGFR-beta) protein-tyrosine kinase, and it is found in more than 90% of DFSP. It seems to play a pivotal role in promoting tumour cell growth. Such a mechanism provides the rationale for the use of imatinib mesylate (IM) and other tyrosine kinase inhibitors (TKIs) in treating advanced DFSP [50].

This tumour is characterized by an infiltrative growth pattern and a high tendency of local recurrence (LR) after the primary resection [51,52]. However, DFSP has low metastatic potential and rarely causes death [31].

LR rates after surgical resection, as reported in the literature, greatly vary and range from 0 to 40% [53]. Although LR usually occur within three years after the surgical resection of the primary tumour, late recurrences are possible.

The most important factor that has a major impact on clinical outcomes for DFSP is the extent of resection. In fact, this high LR rate has been attributed to the missed excision of tumours that are subclinical and have horizontal finger-like extensions in the skin, as well as infiltration of deeper structures. They are clinically unapparent and evidenced only by microscopic examination of lesion margins. Indeed, on microscopic evaluation, multiple projections of neoplastic cells were found to extend laterally or deep up to 3 cm or more from the main lesion [44]. Therefore, the treatment of choice for DSFP is MMS or WLE with histologically confirmed free margins (R0 resection) [54].

MMS is the more frequently recommended approach for DFSP, particularly in tumours developing in sensitive skin areas or for recurrent DSFPs [55]. In MMS, tissue layers are surgically removed sequentially and examined under a microscope during the surgery to define the extent of tumour invasion. Sequential layers are removed until the neoplasm is completely removed. When the tissue sections have been cryostat-frozen, it may be challenging for the pathologist to distinguish the scattered malignant spindle cells from normal fibroblasts. A variant of MMS, named Slow-Mohs, is commonly used. In this MMS variant, tissue samples are not analysed extemporaneously but are fixed and paraffin embedded for a subsequent histological and immunohistochemical evaluation [56,57,58]. However, MMS and its variants are a time-consuming technique, and not widely diffused as standard surgery procedure.

For such reasons, several authors have proposed WLE (with at least 3 cm including the underlying fascia) as a valid therapeutic option to MMS [59].

In some cases, such as facial DFSP, the optimal wide surgical margins of 3 cm are limited by anatomical structures. Postoperative radiotherapy should be considered in cases of close and positive resection margins, but also in cases of several recurrences, in which additional surgical resections could result in potentially disfiguring or functional sequelae [40,41].

The value of RT in the management of DFSP has been mainly assessed in retrospective series [60].

Recently, a series reporting 184 patients highlighted that a free margin <2 cm, as well as the presence of multiple lesions, are both strong predictors of recurrence after a surgical approach and, in both cases, it is advisable to consider adjuvant RT [33].

A smaller analysis coming from another institution was able to identify the initial tumour size >5 cm, the presence of multiple recurrence or of high-grade sarcomatous changes to be clinically relevant indications for adjuvant RT [61].

Considering the salvage setting, which may include either microscopic residual (R1) or macroscopic residual (R2) tumour, RT should be taken into account in all cases where an additional surgery stage could be potentially disfiguring or leading to functional sequelae.

When analysing, in detail, the doses and fractionations schedules, the vast majority of authors use a conventional fractionation (2 Gy/die) with dose ranging from 50 up to 70 Gy in their clinical practice. The clinical target volume should consider the primary anatomical location of the tumour and size of the surgical scar with an additional margin of 3–5 cm, according to the constraints of the surrounding organ at risk.

Considering all the papers reported in this systematic review, only a very few articles had their authors reported adverse events and, in addition, the great variety of the primary anatomical sites involved, which further contributes to the difficulty of providing a comprehensive assessment of the side effects related to adjuvant radiotherapy treatment in DFSP [62].

There are several limitations to our review, in fact, most of the available evidence comes from retrospective series; there are only a few series that include a considerable number of patients. In some series, it is not possible to extrapolate doses and fractionations used for the postoperative RT; in many series, the clinical indication for postoperative RT if adjuvant (presence of risk factors) or salvage (positive margins); in several reports, the number of surgical procedures before postoperative RT is not accurately described; additionally, most data come from single institutions studies.

Prospective larger and multidisciplinary studies are desirable, for all of these reasons, in order to better elucidate these points and improve the clinical knowledge about the role of postoperative RT in DFSP.

## 5. Conclusions

After primary surgical management, postoperative RT may either be considered as adjuvant treatment (presence of risk factors, i.e., close margins, recurrent tumours, aggressive histological subtypes) or as salvage treatment (positive margins), and should be assessed within the frame of multidisciplinary evaluation.

## Figures and Tables

**Table 1:** Features of patients and relative treatment details.

Author	Country	N of Patients	Mean Age (Yrs)	Gender (Male/ Female)	Initial Site	Largest Size (cm)	Prior Surgery	Postoperative Radiotherapy	Indication for RT	Doses	Local Control	Median Follow-Up (Months)
Dai et al., 2023 [1]	China	49	48	92.7%/7.3%	H&N 100%	11	100%	16.3%	A/S	60 Gy	100%	85
Mareş et al., 2022 [27]	Romania	7	38	57.1%/42.9%	Trunk 71.4% Extremities 28.6%	>5	100%	42.9%	A	50–60 Gy	n.a.	12
Wang et al., 2020 [17]	China	6	30	16.7%/83.3%	Trunk 100%	3	100%	33.3%	A	n.a.	100%	36
Du et al., 2019 [33]	China	184	41	76.1%/23.9%	H&N 17.4% Trunk 71.7% Extremities 10.9%	20	100%	23.9%	A/S	50–66 Gy	94.6% *	58
Tsai et al., 2014 [22]	Taiwan	13	11	46.2%/53.8%	H&N 7.7% Trunk 69.2% Extremities 23.1%	8	100%	23.1%	A/S	n.a.	100%	129
Williams et al., 2014 [34]	USA	14	42	50%/50%	H&N 57.1% Trunk 7.2% Extremities 35.7%	12	92.9%	92.9%	A	56–66 Gy	85.7%	126
Hamid et al., 2013 [35]	India	36	38	72.2%/27.8%	H&N 8.3% Trunk 58.4% Extremities 33.3%	20	83.3%	83.3%	A	65–70 Gy	80%	68
Uysal et al., 2013 [36]	Turkey	28	26	64.3%/35.7%	H&N 14% Trunk 22% Extremities 64%	8.4	89.3%	89.3%	A/S	50–70 Gy	89.6% **	81
Castle et al., 2013 [37]	USA	53	41	57%/43%	H&N 36% Trunk 36% Extremities 28%	25	100%	87%	A/S	60–66 Gy	90%	78
Palmerini et al., 2012 [38]	Italy	40	39	55%/45%	Trunk 40% Extremities 60%	n.a.	90%	27%	A	n.a.	77%	49
Fields et al., 2011 [31]	USA	244	42	50%/50%	H&N 14% Trunk 34% Extremities 52%	>5	100%	5.7%	S	n.a.	100%	50
Archontaki et al., 2010 [39]	Greece	16	41	43.6%/56.4%	H&N 31.2% Trunk 56.3% Extremities 12.5%	22.5	100%	18.75%	A	n.a.	100%	44
Heuvel et al., 2010 [40]	The Netherlands	38	38	65.8%/34.2%	H&N 16.7% Trunk 66.6% Extremities 16.7%	22	100%	21%	A/S	50–70 Gy	87.5%	89
Dagan et al., 2005 [41]	USA	10	39	50%/50%	H&N 60% Trunk 10% Extremities 30%	9	100%	100%	A/S	60–65 Gy	90%	95
DuBay et al., 2004 [42]	USA	62	42	37%/63%	H&N 24% Trunk/Extremities 76%	10	100%	5%	S	n.a.	100%	53
Sun et al., 2000 [43]	Taiwan	35	37	68.6%/31.4%	H&N 17.1%. Trunk 60% Extremities 22.9%	25	100%	28.5%	S	46–68 Gy	81.8%	50
Stojadinovic et al., 2000 [44]	USA	33	39	48.5%/51.5%	H&N 100%	8	100%	12.1%	S	60–66 Gy	75%	82
Mentzel et al., 1998 [26]	USA	41	48	54%/46%	H&N 12% Trunk 63% Extremities 25%	n.a.	100%	7.4%	A	n.a.	n.a.	90
Ballo et al., 1998 [45]	USA	19	40	63.2%/36.8%	H&N 36.8% Trunk 42.1% Extremity 21%	15	100%	100%	A/S	50–66 Gy	94.7%	72
Haas et al., 1997 [46]	The Netherlands	38	39	42.1%/57.9%	H&N 13% Trunk 44% Extremities 33%	n.a.	100%	44.7%	A/S	50–66 Gy	82.4%	68
Suit et al., 1996 [47]	USA	18	46	61.1%/38.9%	H&N 55.5% Trunk 27.8% Extremities 16.7%	10	83.3%	66.6%	A/S	50–67 Gy	83.3%	86
Marks et al., 1989 [48]	USA	10	44.4	60%/40%	H&N 50% Trunk 40% Extremities 10%	8.5	70%	70%	A/S	60–67 Gy	90%	12

Legend. * = disease-free survival; ** = relapse-free survival. Abbreviations: N = Number; yrs: Years; H&N = Head and neck; RT = Radiotherapy; A = Adjuvant; S = Salvage; n.a. = Not available.

## Data Availability

Data sharing is not applicable.
